# Viable Compositional Analysis of an Eleven Species Oral Polymicrobial Biofilm

**DOI:** 10.3389/fmicb.2016.00912

**Published:** 2016-06-10

**Authors:** Leighann Sherry, Gillian Lappin, Lindsay E. O'Donnell, Emma Millhouse, Owain R. Millington, David J. Bradshaw, Alyson S. Axe, Craig Williams, Christopher J. Nile, Gordon Ramage

**Affiliations:** ^1^Infection and Immunity Research Group, Glasgow Dental School, School of Medicine, College of Medical, Veterinary and Life Sciences, University of GlasgowGlasgow, UK; ^2^Institute of Healthcare Policy and Practice, School of Health, Nursing and Midwifery, University of the West of ScotlandPaisley, UK; ^3^Strathclyde Institute of Pharmacy and Biomedical Sciences, University of StrathclydeGlasgow, UK; ^4^Gum Health and Dry Mouth Group, GlaxoSmithKline Consumer HealthcareWeybridge, UK

**Keywords:** biofilm, polymicrobial, viability, denture, oral

## Abstract

**Purpose:** Polymicrobial biofilms are abundant in clinical disease, particularly within the oral cavity. Creating complex biofilm models that recapitulate the polymicrobiality of oral disease are important in the development of new chemotherapeutic agents. In order to do this accurately we require the ability to undertake compositional analysis, in addition to determine individual cell viability, which is difficult using conventional microbiology. The aim of this study was to develop a defined multispecies denture biofilm model *in vitro*, and to assess viable compositional analysis following defined oral hygiene regimens.

**Methods:** An *in vitro* multispecies denture biofilm containing various oral commensal and pathogenic bacteria and yeast was created on poly (methyl methacrylate) (PMMA). Denture hygiene regimens tested against the biofilm model included brushing only, denture cleansing only and combinational brushing and denture cleansing. Biofilm composition and viability were assessed by culture (CFU) and molecular (qPCR) methodologies. Scanning electron microscopy and confocal laser scanning microscopy were also employed to visualize changes in denture biofilms following treatment.

**Results:** Combinational treatment of brushing and denture cleansing had the greatest impact on multispecies denture biofilms, reducing the number of live cells by more than 2 logs, and altering the overall composition in favor of streptococci. This was even more evident during the sequential testing, whereby daily sequential treatment reduced the total and live number of bacteria and yeast more than those treated intermittently. Bacteria and yeast remaining following treatment tended to aggregate in the pores of the PMMA, proving more difficult to fully eradicate the biofilm.

**Conclusions:** Overall, we are the first to develop a method to enable viable compositional analysis of an 11 species denture biofilm following chemotherapeutic challenge. We were able to demonstrate viable cell reduction and changes in population dynamics following evaluation of various denture cleansing regimens. Specifically, it was demonstrated that daily combinational treatment of brushing and cleansing proved to be the most advantageous denture hygiene regimen, however, residual organisms still remained within the pores of PMMA surface, which could act as a reservoir for further biofilm regrowth. We have identified an industry need for denture cleansing agents with the capacity to penetrate these pores and disaggregate these complex biofilm consortia.

## Introduction

Denture stomatitis (DS) is characterized as the erythema and inflammation of the oral mucosa, localized under dentures. Although *Candida albicans* can be present in the oral cavity of up to 75% of the healthy population (Arendorf and Walker, 1987; ten Cate et al., 2009; Singh et al., 2014), it is an opportunistic pathogen and has been well-established as the main causative agent of DS (Barbeau et al., 2003; Jose et al., 2010; Gendreau and Loewy, 2011). The presence of *C. albicans* in the oral cavity is reliant on a number of factors, including but not limited to; ill-fitting dentures, smoking, breach of host defenses and antibiotic use (Salerno et al., 2011; Kraneveld et al., 2012; O'Donnell et al., 2015a). Although the majority of research has focused around *C. albicans* being the primary causative microbial agent in DS, recent data also indicates that 10-fold more bacteria than yeasts are observed on denture surfaces (Teles et al., 2012; O'Donnell et al., 2015a).

There has been growing interest surrounding how fungal-bacterial interactions in the oral cavity influence disease (Sumi et al., 2002; Ealla et al., 2013; O'Donnell et al., 2015a). To this end, denture biofilm systems have been developed to model and test polymicrobial infections, however, these tend to be limited to 2-3 organisms, or rely on undefined inocula from clinical samples. Ultimately this makes it difficult to reproduce and fully understand the impact of multi-species biofilm consortia in denture patients (Coulthwaite and Verran, 2008; Li et al., 2010; Urushibara et al., 2014). Therefore, there is the need for the development of a defined *in vitro* multi-species denture biofilm, as this would provide a greater understanding to clinically relevant polymicrobial oral diseases and the treatment of these using various denture regimens.

Poly(methyl) methacrylate (PMMA) is the main choice of denture material used clinically, however, the uneven surface results in areas of depression that provides *C. albicans* and other organisms the ideal surface to form biofilms and evade denture cleansing therapies (Li et al., 2010; Ramage et al., 2012; Mendonca E Bertolini et al., 2014). Various physical and chemical cleansing techniques both individually and in combination have been investigated with regards to denture hygiene in order to determine the optimal method for cleaning. However, most of these techniques evaluate treatment over a short period of time and therefore do not simulate regular daily denture cleaning routines (Pavarina et al., 2003; Felton et al., 2011; Pellizzaro et al., 2012). The impact of daily denture cleansing treatment has been investigated previously, and despite a significant reduction of viable *C. albicans* cells initially, residual yeast cells were still present within the biofilm that could proliferate if treatment was not completely effective and allow regrowth of the organism (Ramage et al., 2012; Faot et al., 2014; Freitas-Fernandes et al., 2014). A caveat to these studies was that they used models consisting of only one organism, which is not reflective of the denture microenvironment. Furthermore, using culture techniques as the sole source of viability testing may not prove to be the most reliable method, with studies identifying various bacteria and yeasts that can enter a “viable but non-cultivable” state upon stress (Divol and Lonvaud-Funel, 2005; Oliver, 2005). Moreover, the complex composition of these microbial communities hinders the ability of conventional microbiology to sensitively quantify and qualify the organisms present. Therefore, alternative molecular approaches may prove to be more sensitive and specific when assessing viability of biofilms.

The aims of the present study were to develop a multispecies biofilm model that was representative of a DS environment and to devise a rapid and sensitive method to quantify the viable composition of biofilms challenged with either monotherapy or combinational denture cleansing regimens. The overall aim was to test these methods to determine which had the greatest impact on biofilm viability and disruption.

## Materials and methods

### Growth and standardization of bacteria

A selection of laboratory strains of microorganisms associated with denture biofilms were used in this study for the construction of a denture biofilm model, based on our own and previously published studies (Sachdeo et al., 2008; Malcolm et al., 2016). These included *Streptococcus mitis* NCTC 12261*, Streptococcus intermedius* ATCC 27335*, Streptococcus oralis* ATCC 35037 and *Aggregatibacter actinomycetemcomitans* OSM 1123, which were grown and maintained at 37°C on Colombia blood agar (CBA [Oxoid, Hampshire, UK]) in 5% CO_2_. *C. albicans* 3153A which was maintained on Sabouraud's dextrose agar (Oxoid) at 30°C for 48 h. *Fusobacterium nucleatum* ATCC 10596, *F. nucleatum* ssp. *vincentii* ATCC 49256*, Actinomyces naeslundii* ATCC 19039*, Veillonella dispar* ATCC 27335*, Prevotella intermedia* ATCC 25611 and *Porphyromonas gingivalis* W83 which were maintained at 37°C on fastidious anaerobic agar (FAA [Lab M, Lancashire, UK]) in an anaerobic incubator (Don Whitley Scientific Limited, Shipley, UK) with an atmosphere of 85% N_2_, 10% CO_2_ and 5% H_2_.

Overnight broths of *S. mitis, S. intermedius, S. oralis* and *A. actinomycetemcomitans* were grown in tryptic soy broth (TSB, Sigma-Aldrich, Dorset, UK) supplemented with 0.6% w/v yeast extract (Formedium, Hunstanton, UK) and 0.8% w/v glucose (Sigma-Aldrich). *C. albicans* was grown in yeast peptone dextrose (YPD, Sigma-Aldrich) for 18 h at 30°C. *P. gingivalis, F. nucleatum, F. nucleatum* ssp. *vincentii* were propagated in 10 mL Schaedler's anaerobic broth (Oxoid) and *V. dispar, A. naeslundii* and *P. intermedia* were grown in 10 mL of brain heart infusion (BHI, Sigma-Aldrich) broth. Cultures were grown for 24–48 h at 37°C as necessary, washed by centrifugation and resuspended in phosphate buffered saline (PBS, Sigma-Aldrich). All cultures were standardized and adjusted to a final working concentration of 1 × 10^7^ cells/mL for downstream biofilm studies.

### Development of denture biofilm model

Biofilms were formed in a similar sequential approach to our previous studies (Sherry et al., 2013; Millhouse et al., 2014). Briefly, standardized *S. mitis, S. intermedius, S. oralis*, and *C. albicans* in artificial saliva (AS), were added to a 1 cm diameter poly (methyl methacrylate) disc (PMMA, Chaperlin and Jacobs Ltd, Surrey, UK) contained within a 24 well plate (Corning, NY, USA). AS components included porcine stomach mucins (0.25% w/v), sodium chloride (0.35% w/v), potassium chloride (0.02 w/v), calcium chloride dihydrate (0.02% w/v), yeast extract (0.2% w/v), lab lemco powder (0.1% w/v), proteose peptone (0.5% w/v) in ddH_2_O (Sigma-Aldrich). Urea was then added independently to a final concentration of 0.05% (v/v). The plate was then incubated at 37°C in 5% CO_2_ for 24 h, adapted from a method previously described (Millhouse et al., 2014).

Following incubation, the supernatant was removed and standardized *F. nucleatum, F. nucleatum* ssp. *vincentii, A. naeslundii*, and *V. dispar* were added to the biofilms and incubated at 37°C anaerobically for 24 h. Finally, standardized *P. gingivalis, P. intermedia*, and *A. actinomycetemcomitans* were added to the PMMA discs already containing the previous 8 microorganisms. Biofilms were incubated at 37°C anaerobically for a further 4 days, with spent supernatants removed and replaced with fresh AS daily. The 11 species biofilms were then stored at –80°C until required.

### Treatment of complex denture biofilms

Following biofilm development, each disc was gently washed with 1 mL of PBS to remove any non-adherent cells. Treatment with denture cleanser Polident, Sub-brand name (GlaxoSmithKline Consumer Healthcare, Surrey, UK) mimicked pack use instructions. PMMA discs containing multispecies biofilms were placed in a sterile beaker containing 150 mL of 375 ppm hard water (HW) at 40°C before the denture tablet was added, initiating treatment. After 3 min PMMA discs were removed from the beaker and placed in a 24 well plate containing 1 mL of Dey-Engley neutralizing broth (Sigma-Aldrich) and incubated for 15 min anaerobically. This ensured complete inactivation of the compound before microbiological analysis. Untreated controls were maintained in 1 mL HW during the treatment stage and blanks containing no inoculum were also included.

For brushing treatments, PMMA discs containing the complex biofilm were brushed 5 times across the surface in HW using a toothbrush. This was based on the surface area and average time of denture brushing, as previously described (Ramage et al., 2012). For combinational treatment, brushing with HW was carried out either before or after DC treatment (3 min). PMMA discs were then neutralized as described previously before microbiological analysis was undertaken. Testing was carried out in triplicate and on three separate occasions, for all denture cleaning regimens.

### Biofilm viability analysis by colony forming units (CFU)

CFU analysis was performed as a measure of how active each treatment was against the complex denture biofilms. Following treatment and neutralization, PMMA discs were sonicated at 35 kHz for 10 min to remove the biomass, as previously described (Ramage et al., 2012) before the Miles and Misra technique was employed (Miles et al., 1938). Serial dilutions were plated on BHI + 10% blood plates and incubated aerobically and anaerobically at 37°C for 48 h. In addition, samples were also plated on SAB agar and incubated at 30°C for 48 h. The number of colonies were counted and represented as total aerobes, total anaerobes and total yeast.

### Differentation of total and live cells within biofilms

Viability of the treated biofilms was also assessed using live dead PCR in order to enumerate the definitive and relative composition of the biofilms, a technique that has been shown to differentiate viable and dead cells from various oral bacteria biofilms (Alvarez et al., 2013; Sanchez et al., 2013; Sánchez M. C. et al., 2014). This method is based upon propidium monoazide (PMA), a DNA-intercalating dye that is able to bind to DNA following exposure to a halogen light source (Nocker et al., 2006). Binding can only occur in dead cells or those with compromised membrane integrity as PMA is unable to permeablise cell membranes (Sánchez M. C. et al., 2014). This covalent bonding prevents downstream amplification in quantitative PCR (qPCR) and therefore only live cells can be detected.

Samples were prepared as previously described by Sanchez et al., with some modifications (Sánchez M. C. et al., 2014). In brief, sonicated samples had 50 μM of PMA added to each sample and incubated in the dark for 10 min to allow uptake of the dye. Samples were then exposed to a 650 W halogen light for 5 min before DNA was extracted using the QIAamp DNA mini kit, as per manufacturer's instructions (Qiagen, Crawley, UK). No PMA controls were also included for each sample to determine total biomass. The extracted DNA underwent quality checks using the NanoDrop spectrophotometer (Fisher Scientific, Loughborough). Samples with a 260/280 nm ratio of 1.8 to 2.2 were deemed to be of high quality and used in subsequent PCR experiments.

### Quantitative analysis of biofilm composition

Real-time quantitative PCR (qPCR) was performed to determine the live and total cells remaining in the biofilm following each treatment. Briefly, 1 μL of extracted DNA was added to a mastermix containing 12.5 μL SYBR® GreenER™ (Life Technologies, Paisley, UK), 9.5 μL UV-treated RNase-free water and 1 μL of 10 μM forward/reverse primers for each bacterial/fungal species. The primers used were previously published and are listed in Table 1. The thermal profile used consisted of an initial denaturation of 95°C for 10 min followed by 40 cycles of 30 s at 95°C, 60 s at 55°C, and 60 s at 72°C. For *C. albicans*, 16S and 18S primer sets, the annealing temperature of 60°C was used. Three independent replicates from each parameter were analyzed in triplicate using MxProP Quantitative PCR machine and MxPro 3000P software (Stratagene, Amsterdam, Netherlands). Samples were quantified to calculate the colony forming equivalent (CFE) based upon a previously established standard curve methodology of bacterial colony forming units ranging from 1 × 10^3^ to 10^8^ CFU/mL (O'donnell et al., 2015b). Melting curve analysis was performed for all primer sets to ensure a single peak, which was indicative of primer specificity.

**Table 1:** **Bacterial and fungal primers for real time qPCR**.

**Primer**	**Sequence (5′–3′)**	**References**
A. A^*^	F—GAACCTTACCTACTCTTGACATCCGAA R—TGCAGCACCTGTCTCAAAGC	Loozen et al., 2011
*A. naeslundii*	F—GGCTGCGATACCGTGAGG R—TCTGCGATTACTAGCGACTCC	Periasamy et al., 2009
*C. albicans*	F—GGGTTTGCTTGAAAGACGGTA R—TTGAAGATATACGTGGTGGACGTTA	This study
*F. nucleatum*	F—GGATTTATTGGGCGTAAAGC R—GGCATTCCTACAAATATCTACGAA	Sherry et al., 2013
*P. intermedia*	F—CGGTCTGTTAAGCGTGTTGTG R—CACCATGAATTCCGCATACG	Loozen et al., 2011
*P. gingivalis*	F—GGAAGAGAAGACCGTAGCACAAGGA R—GAGTAGGCGAAACGTCCATCAGGTC	Park et al., 2011
*V. dispar*	F—CCGTGATGGGATGGAAACTGC R—CCTTCGCCACTGGTGTTCTTC	Periasamy and Kolenbrander, 2009
*Streptococcus*	F—GATACATAGCCGACCTGAG R—CCATTGCCGAAGATTCC	Sherry et al., 2013
16S	F—CGCTAGTAATCGTGGATCAGAATG R—TGTGACGGGCGGTGTGTA	Suzuki et al., 2004b
18S	F—CTCGTAGTTGAACCTTGGGC R—GGCCTGCTTTGAACACTCTA	Rajendran et al., 2015

* *A. actinomycetemcomitans*.

### Sequential denture cleaning techniques

**Figure 1:**
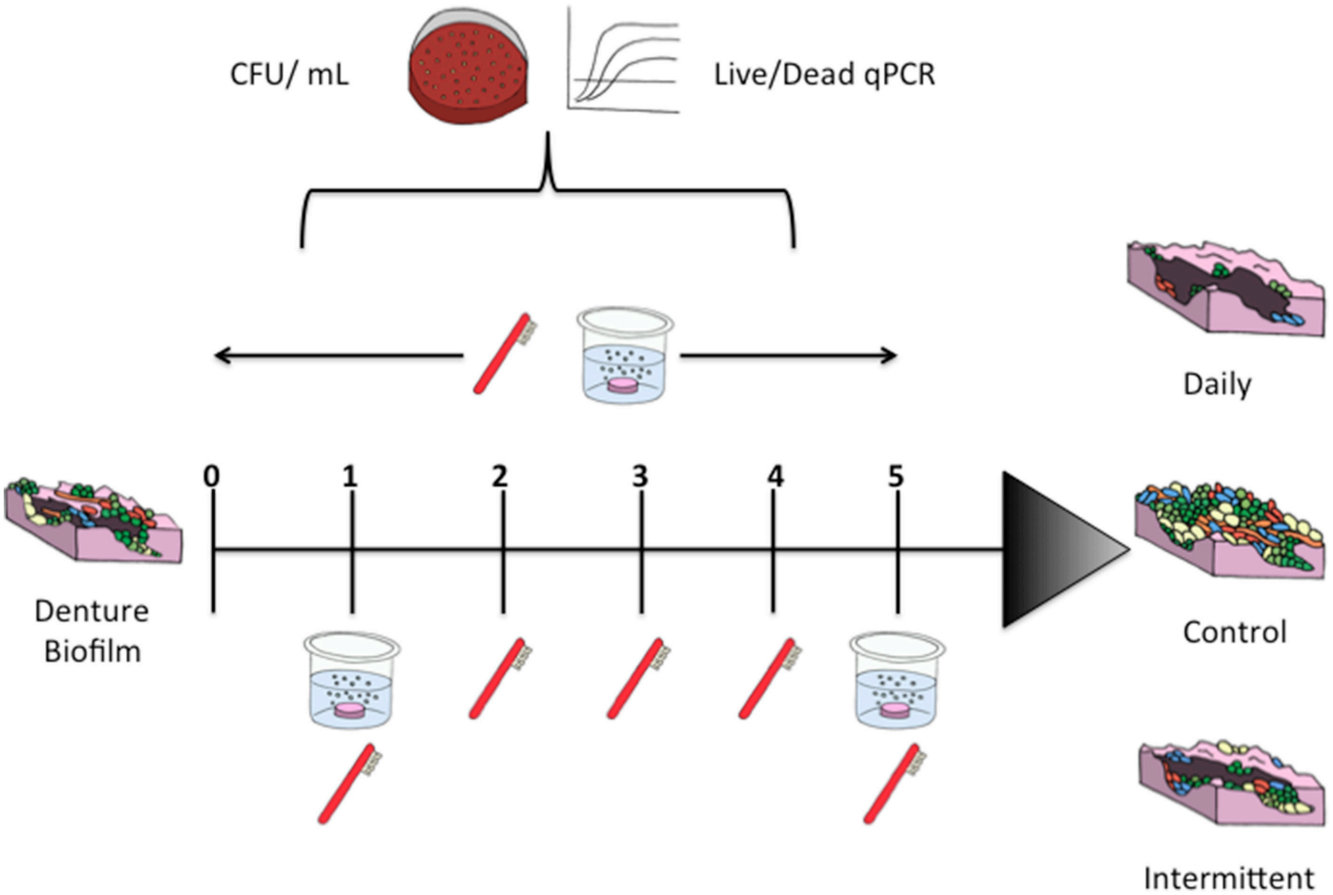
**Sequential treatment of denture biofilm protocol**. PMMA discs were placed in 24 well plates for biofilm culture. Biofilms were treated daily with brushing and a denture cleanser for 5 days or were brushed every day with denture cleansing on day 1 and day 5. Untreated controls were maintained in artificial saliva during treatments.

To investigate whether sequential combinational denture cleansing techniques were more advantageous than intermittent treatment, multispecies biofilms were treated daily over the course of 5 days, as illustrated in Figure 1. Treatments were either combinational therapy of brushing with HW followed by a 3 min DC for 5 consecutive days or daily brushing with intermittent DC on day 1 and day 5 only.

Following each treatment, discs were incubated in Dey-Engley neutralizing broth for 15 min in the anaerobic chamber, before being incubated in artificial saliva within the anaerobic chamber until the next treatment time. Untreated biofilms were maintained in HW during each treatment time and served as positive controls. Antimicrobial activity was assessed by CFU and CFE, as described above.

### Ultrastructural changes of multispecies biofilms

Scanning electron microscopy (SEM) was performed on 11 species biofilms grown on PMMA discs. Following maturation biofilms were carefully washed with PBS before their respective treatments were employed, as described above. Biofilms were then carefully washed twice with PBS and then fixed in 2% (v/v) para-formaldehyde, 2% (v/v) glutaraldehyde and 0.15 M sodium cacodylate, and 0.15% w/v Alcian Blue, pH 7.4, and prepared for SEM as previously described (Erlandsen et al., 2004; Sherry et al., 2012). The specimens were sputter-coated with gold and viewed under a JEOL JSM-6400 scanning electron microscope. Images were assembled using Photoshop software (Adobe, San Jose, CA, USA).

In addition to SEM, confocal microscopy was used to visualize the presence of live bacteria following treatment regimen. Following treatment and neutralization of biofilms, cells were stained using the LIVE/DEAD® BacLight™ bacterial viability kit (Fisher Scientific, Leicestershire, UK) containing SYTO9 and propidium iodide (PI). These dyes were used in a 1:1 combination with 1 mL being added to each PMMA disc containing biofilms and stained for 15 min in the dark at 37°C. Biofilms were then washed with 1 mL of PBS and fixed with 2% para-formaldehyde (PFA) for 1 h. PMMA discs were washed in PBS for a final time and mounted to glass slides for viewing under a confocal laser scanning microscope (CLSM [Leica SP5]), at excitation and emission wavelengths, respectively, of 488/500 nm for SYTO9 and 532/635 nm for PI. One representative biofilm from each group was digitally photographed.

### Statistical analysis

Data distribution, graph production and statistical analysis were performed using GraphPad Prism (version 5; La Jolla, CA, USA). After assessing whether data conformed to a normal distribution, One-way Analysis of Variance (ANOVA) and *t* tests were used to investigate significant differences between independent groups of data that approximated to a Gaussian distribution. A Bonferroni correction was applied to the *p* value to account for multiple comparisons of the data. Any non-parametric data was analyzed using the Mann-Whitney U-test or the Kruskal-Wallis test with a Dunn's post-test to assess differences between independent sample groups. Statistical significance was achieved if *P* < 0.05.

## Results

### Quantitative analysis of a multi-species denture biofilm model

**Figure 2:**
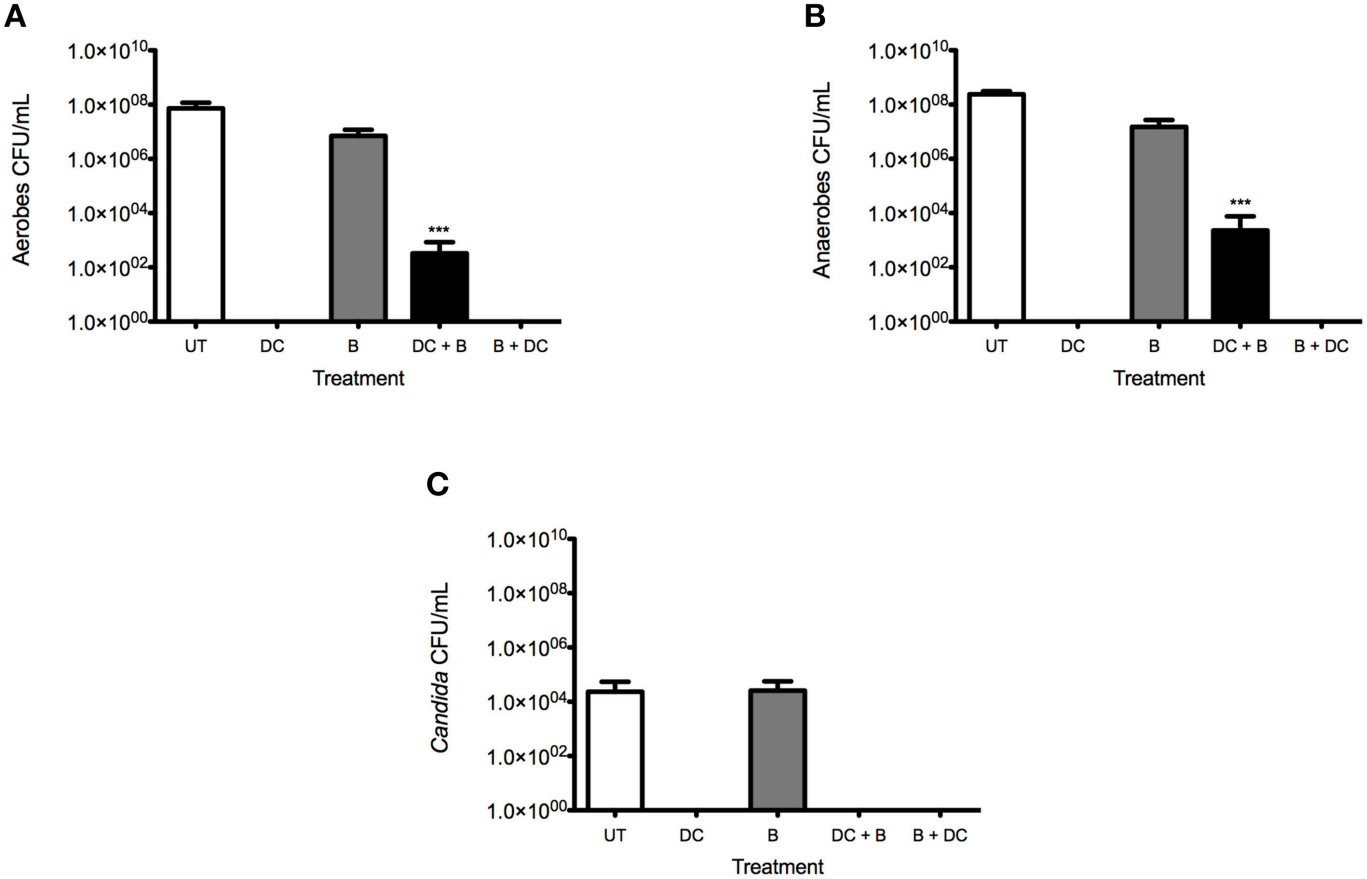
**Multi-species biofilm viability is greatly impacted by combinational treatment compared to monotherapy**. Multispecies biofilms were grown on PMMA for 7 days, as previously described. Following maturation, biofilms were washed and either treated with a denture cleanser (DC) for 3 min, brushed only **(B)**, exposed to a combinational treatment of brushing before denture cleansing (B + DC) or brushing after denture cleansing (DC + B). Viability of total aerobes **(A)**, anaerobes **(B)** and *Candida* **(C)** was assessed by CFU counts. Untreated (UT) controls were also included. All testing was carried out in triplicate and on three independent occasions. Data represents mean ± SD, statistical analysis of treatments were compared to the untreated control (^***^p < 0.001).

Multi-species biofilms treated with various denture-cleansing regimens were initially quantified by CFU for total aerobes (Figure 2A), anaerobes (Figure 2B), and yeast (Figure 2C). It was evident that all techniques with the exception of brushing only had significantly reduced CFUs. Brushing alone was only able to reduce the number of total aerobes from 7.3 × 10^7^ CFU/mL to 7.0 × 10^6^ CFU/mL and total anaerobes from 2.4 × 10^8^ CFU/mL to 1.5 × 10^7^ CFU/mL. Interestingly, there was a slight increase in the number of yeast cells following treatment, from 2.3 × 10^4^ CFU/mL to 2.6 × 10^4^ CFU/mL. However, when the combinational treatment of DC and brushing (DC + B) was used, total aerobes and anaerobes were reduced to 3.3 × 10^2^ and 2.3 × 10^3^ CFU/mL (*P* < 0.0001), respectively.

This is in contrast to DC monotherapy and B + DC treatment, whereby no growth was observed. The discrepancies between these cleansing regimens is thought to be a result of the DC only having an effect on the most upper layers of the biofilm, therefore when brushing is applied following DC, the physical disruption of the biomass removes this outer layer and exposes live cells that may be colonizing the crevices of the PMMA surface. No *Candida* were detected with DC + B. Both DC only and brushing followed by DC (B + DC) showed the greatest reduction of aerobic and anaerobic organisms, with no CFU observed.

**Figure 3:**
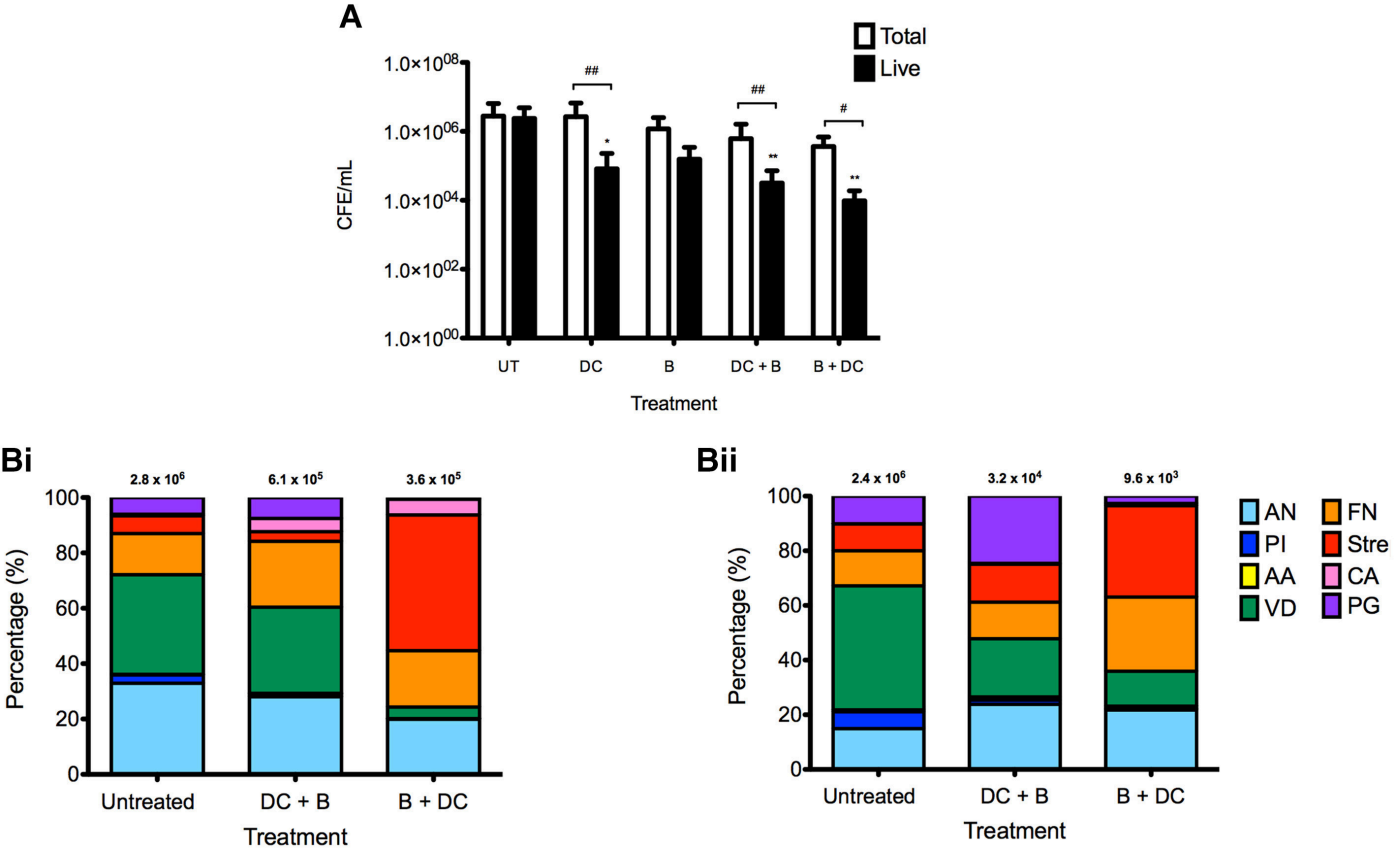
**Biofilm compositional analysis of denture biofilms following oral hygiene regimens**. Multispecies biofilms were grown on PMMA for 7 days before treated with the four therapies; denture-cleansing (DC), brushing (B), cleansing then brushing (DC + B) and brushing then cleansing (B + DC). Following treatment, each disc was sonicated before 50 μM of PMA was added and exposed to a 650 w halogen light source for 5 min to allow photo activation. Samples containing no PMA were also included to account for total biomass. DNA was extracted from each sample using the Qiagen DNA extraction kit, for quantification of each species using SYBR® GreenER™ based qPCR to determine the number of total and live cells remaining following treatment **(A)**. The composition of the biofilms following combinational treatment was also determined using species-specific primers **(B)** with total **(i)** and live **(ii)** cells shown. All testing was carried out in triplicate and on three independent occasions. Data represents mean ± SD, statistical analysis of treatments was compared to their respective untreated controls, in addition to total vs. live for each therapy (^*^/^#^*p* < 0.05, ^**^/^*##*^*p* < 0.01).

Despite these findings, the survival of these microbes was further assessed using qPCR, as this is deemed as a more sensitive technique for quantification. Initially, all species-specific data were combined to show the overall trend with each treatment tested (Figure 3A). The most superior treatments in terms of biofilm biomass and viability reduction were combinational therapies. B + DC reduced the total biomass by 87% from 2.8 × 10^6^ CFE/mL to 3.6 × 10^5^ CFE/mL. Furthermore, of the total biomass remaining following treatment the number of live cells was significantly reduced, with only 2.7% (9.6 × 10^3^ CFE/mL) cells remaining (*P* = 0.0237), compared to 2.4 × 10^6^ CFE/mL of the live cells in the untreated control. Combinational treatment of DC + B had the second optimal reduction of microbes with 22% (6.1 × 10^5^ CFE/mL) of the biofilm remaining following treatment. Of this, only 5.1% (3.2 × 10^4^ CFE/mL) of the biofilm represented live cells (*P* = 0.0064).

Although DC monotherapy was only able to reduce biofilm biomass by 4% (2.6 × 10^6^ CFE/mL), it was able to effectively kill 97% (8.2 × 10^4^ CFE/mL) of the remaining biomass (*P* = 0.0044). In addition, although brushing alone was able to reduce the total biofilm biomass to 42% (1.2 × 10^6^ CFE/mL), this cleansing method had the least impact on the biofilm with regards to live cells, of which 13% (1.6 × 10^5^ CFE/mL) remained following brushing only.

Next, individual species were investigated to determine if combinational treatments had a greater impact on specific species composition compared to others (Figure 3B). Compositional analysis of the combinational therapies was investigated as these regimens proved to be more superior than montherapies shown in previous figures. Initial analysis was undertaken to show the percentage of total (Figure 3Bi) and live (Figure 3Bii) cells with the untreated and treated biofilms. Of particular interest, the proportion of *A. naeslundii* and *V. dispar* in the biofilm was reduced from 32 and 36% to 28 and 31%, respectively, when treated with DC + B. These bacteria were reduced further when B + DC was employed, with 19 and 4% of the biofilm composed of *A. naeslundii* and *V. dispar*, respectively. A similar trend was found across all treatment regimens with these organisms (Supplementary Figure 1), however, the number of live cells remaining following combinational treatment was lower than single therapy counterparts. In contrast, *Streptococcus* species made up the majority of the biofilm composition following B + DC, increasing from 6% of the untreated biofilm to 49% following treatment. Moreover, *C. albicans* did not account for a substantial proportion of the untreated biofilm, only accounting for less than 1% of the total biofilm biomass. However, when both treatments were employed, there was a shift in species distribution, allowing *C. albicans* to make up ~5% of the total biomass. Supplementary Table 1i reports the percentage of each species making up the biofilms, pre- and post-treatment.

In addition, when the live cells only were considered (Figure 3Bii), *V. dispar* followed the same pattern as before where the untreated biofilm was made up of 45% of live *V. dispar* cells, which reduced to 21 and 12% with DC + B and B + DC, respectively. Furthermore, 33% of the live cells in the biofilm following B + DC consisted of *Streptococcus* species, early colonizers of the oral cavity. Of particular interest, *C. albicans* only accounted for 6% of the total biofilm remaining following B + DC, with 3% of these cells live. For all species specific changes see Supplementary Figure 1.

### Daily combinational treatment reduces denture biofilm biomass

**Figure 4:**
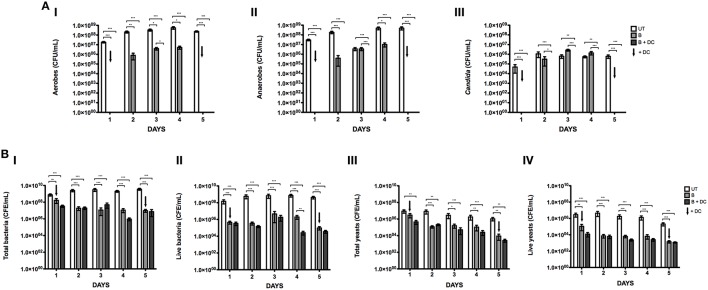
**Daily cleaning of denture biofilms reduces the biofilm biomass and viability**. Multispecies complex biofilms were grown on PMMA for 7 days, as previously described. Following maturation, biofilms were washed and either treated daily with brushing and denture cleansing (B + DC) or brushed daily with the addition of a DC on day 1 and 5 only **(B)**. Viability of total aerobes **(Ai)**, anaerobes **(Aii)**, and *Candida* **(Aiii)** was assessed by CFU. Biofilms were also treated with PMA and exposed to a 650 w halogen light for live-dead PCR analysis. Samples containing no PMA were also included to account for total biomass. DNA was extracted from each sample using the Qiagen DNA extraction kit, for quantification of total **(Bi)** and live **(Bii)** bacteria and total **(Biii)** and live yeast **(Biv)** using SYBR® GreenER™ based qPCR. All testing was carried out in triplicate and on three independent occasions. Data represents mean ± SD, statistical analysis of treatments was compared to their respective untreated controls, in addition to total vs. live for each therapy (^*^p < 0.05, ^**^p < 0.01, ^***^p < 0.0001).

Sequential therapy of B + DC was identified earlier in this study as a superior treatment for denture biofilms, however, this has only been shown from a single cross sectional analysis. Therefore, longitudinal daily sequential treatment of these biofilms over a course of 5 days was investigated and compared to those that were treated with such therapy intermittently. Initial CFU analysis was performed and revealed that untreated biofilms continued to grow and mature over the course of 5 days. Total aerobes increased from 1.8 × 10^7^ to 2.3 × 10^8^ CFU/mL (Figure 4Ai), total anaerobes rose from 3.0 × 10^7^ to 4.9 × 10^8^ CFU/mL (Figure 4Aii) and total yeasts increased from 4.8 × 10^4^ to 6.1 × 10^5^ CFU/mL (Figure 4Aiii), when comparing day 1 to day 5.

Sequential treatment of B + DC was the most effective therapy used over the course of 5 days, as no CFU were recorded for total aerobes, anaerobes and yeast on any day following treatment. However, when denture biofilms were brushed with no subsequent denture cleansing on days 2 to 4, there was regrowth of organisms recorded. By day 4, there was a 2-log reduction (5.2 × 10^6^ CFU/mL) in the number of aerobes present following brushing (*P* < 0.001), compared to the untreated control (5.6 × 10^8^ CFU/mL; Figure 4Ai). A similar finding was also observed for the anaerobes, whereby the total number of organisms remaining post-treatment was ~1.5 log less [1.0 × 10^7^ CFU/mL (*P* < 0.001)] than the untreated control (4.9 × 10^8^ CFU/mL; Figure 4Aii). Although yeast CFU followed the same pattern increasing with intermittent brushing from no CFU to 1.4 × 10^6^ CFU/mL by day 4, there was no reduction compared to the untreated control at this time point.

These results were further investigated at a molecular level using the live/dead PCR assay for detection of total and live bacteria/fungi (Figure 4B). As previously observed in this study, a significant number of bacteria and fungi are detected using qPCR methodologies following both daily and intermittent treatment regimens compared to CFU counts in Figure 4A. Although there were very few significant differences between both therapies, daily B + DC appeared to have the slight advantage over intermittent cleansing against both bacteria and fungi. At day 4, the total number of bacteria present in the biofilm following intermittent cleansing treatment was 12 × greater compared to daily B + DC on the same day (Figure 4Bi). However, on day 5 both treatments proved to be equally active with 9.8 × 10^6^ and 7.4 × 10^6^ CFE/mL remaining after intermittent cleansing and B + DC, respectively. Despite a substantial number of organisms still remaining after 5 days, both treatments significantly reduced the microbial burden of the untreated biofilm from 3.7 × 10^9^ CFE/mL (*P* < 0.0001).

A similar finding was observed in the number of live bacteria post-treatment, with daily B + DC having the greatest impact on each day tested (Figure 4Bii). Of particular interest, daily B + DC treatment resulted in significantly less live cells (~2 logs) compared to intermittent cleansing biofilms (2.0 × 10^6^ CFE/mL, *P* < *0.01*). However, both techniques significantly reduced the overall live bacterial burden from 7.3 × 10^8^ CFE/mL (*P* < 0.0001) on day 4.

On days 3 and 4, the number of total yeasts remaining in the biofilms following daily B + DC was significantly lower than those treated with intermittent cleansing, when each was compared to the untreated control (Figure 4Biii). Intermittent cleansing reduced *C. albicans* to 1.6 × 10^5^(*P* < 0.05) and 1.1 × 10^5^ CFE/mL (*P* < 0.01) on days 3 and 4, respectively, compared to the daily B + DC whereby the burden was reduced to 5.1 × 10^4^CFE/mL (*P* = 0.0003) and 2.6 × 10^4^ CFE/mL (*P* = 0.0003).

The number of live yeast cells was fairly similar between the two treatments, both of which were significantly less than the untreated control on all days (Figure 4Biv). In fact, on day 5 both daily and intermittent cleansing reduced the number of live *C. albicans* remaining in the biofilm by 2 logs to ~1.0 × 10^3^ CFE/mL (*P* < *0.0001*), from 2.1 × 10^5^ CFE/mL in the untreated control.

One finding of interest in this sequential study was the regrowth of organisms at days 2 to 4 when brushing only was used to treat the biofilms, measured by culture methodologies. However, when molecular analysis was undertaken, live cells were persistent on all days of treatment, with no increase in growth detected over the testing period.

### Combinational therapy impacts biofilm architecture

**Figure 5:**
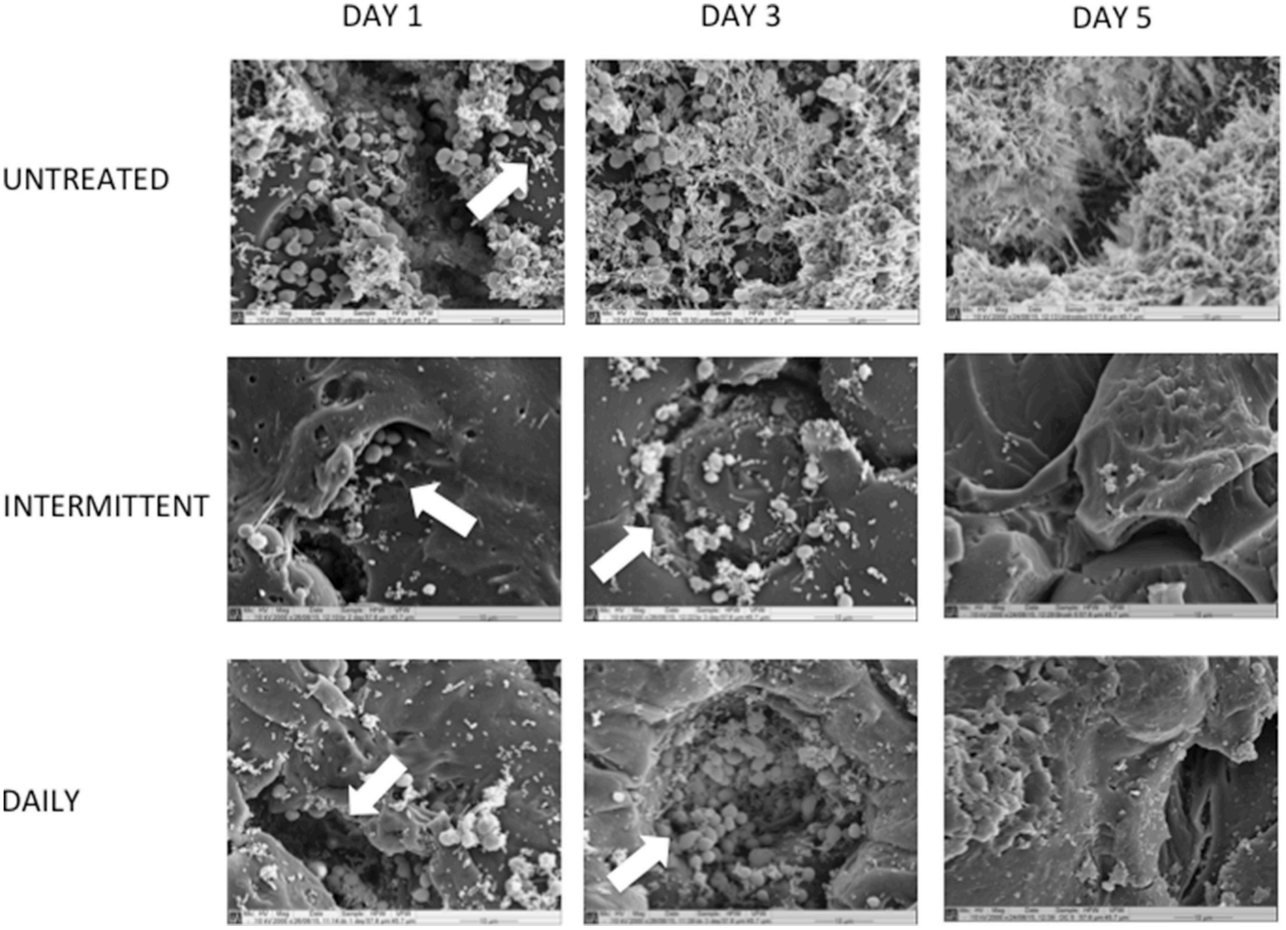
**Daily combinational treatment impacts biofilm architecture by reducing total biomass**. Multispecies biofilms were grown on PMMA for 7 days, as previously described. Following biofilm development, discs were washed and treated with B + DC daily for 5 days or brushed daily with intermittent cleansing on day 1 and 5. Untreated biofilms were also included for comparison. Biofilms were then processed and viewed on a JEOL-JSM 6400 scanning electron microscope and images assembled using Photoshop software. All images are shown at 2000 × magnifications and are representative of the sample. Scale bars represent 10 μm. Note the pores within the PMMA as denoted by arrows.

To determine if these differences in microbial composition affected the denture biofilm architecture, SEM was employed to visualize changes in biomass at day 1, 3, and 5 (Figure 5). At day 1, untreated biofilms were shown to be fairly complex with *C. albicans* yeast cells and *Streptococcus* species appearing to be the dominating species within the biofilm. Following treatment, there was a substantial visible reduction in biofilm biomass compared to the untreated control, with many yeast and streptococci persisting in the crevices of the PMMA material.

As the untreated biofilm continued to grow for a further 2 days, it was visibly evident there was an increase in not only biofilm biomass but based on architecture the distribution of individual species. We also observe that *C. albicans* coaggregates with individual bacterial cells, bringing stability and maturity to the complex biofilm. However, without further detailed species-specific microscopy, such as fluorescent *in situ* hybridization, we cannot say with certainty which species are present. When therapeutic measures were carried out on day 3, the majority of the biofilm seemed to be removed from the surface of the PMMA but deep pores of the material remained full of organisms, as denoted by arrows. Of particular interest, when brushing only was used there was an equal variety of organisms present including rod-shaped bacteria across the surface and in pores. In contrast, daily B + DC appears to not only reduce the majority of biomass, but yeast cells coaggregating with bacteria are evident within the PMMA pores.

On the final day of treatment (day 5), the untreated control was a complex, mature biofilm surrounded by an extracellular matrix, making it difficult to differentiate between individual species. Both treatment regimens were effective at reducing the overall biomass of the biofilm after 5 days.

**Figure 6:**
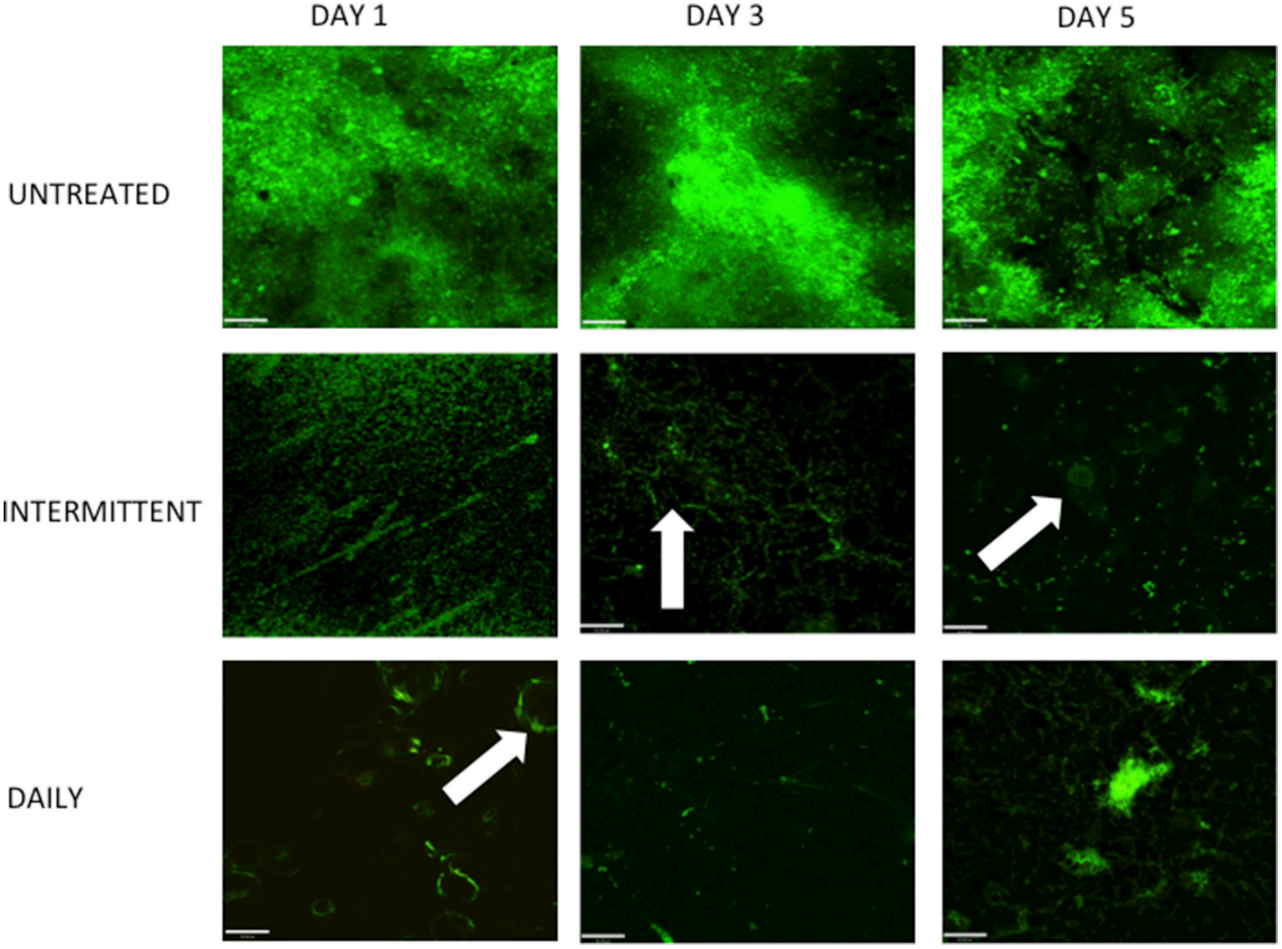
**Live cells imaging reveals viable cells within pores following treatment**. Multispecies biofilms were grown on PMMA for 7 days, as previously described. Following biofilm development, discs were washed and treated with B + DC daily for 5 days or brushed daily with intermittent cleansing on day 1 and 5. Untreated biofilms were also included for comparison. Images were stained with SYTO9 and PI to show live and dead cells remaining following treatment and viewed under a CLSM (Leica SP5). All images are shown at 20 × magnification and scale bars represent 20 μm. Note the pores within the PMMA as denoted by the arrows.

Finally, CLSM was used to visualize the live cells remaining within the denture biofilm following daily and intermittent cleaning (Figure 6). The number of viable cells present in the untreated controls appeared to be constant throughout the 5 days of testing, confirming the data represented in Figure 4B. Both daily and intermittent combinational therapy reduced biofilm biomass substantially compared to the untreated control, at each time point. However, when comparing both treatment regimens to one another there appeared to be minimal differences with regards to viability. Biofilms treated intermittently with DC appeared to have a homogenous distribution of viable cells across the surface of the PMMA, whereas daily B + DC therapy resulted in localized areas of live cells, particularly at day 5.

## Discussion

The development of a multispecies denture biofilm model provides a platform for evaluating various oral hygiene regimens *in vitro*. The inadequate removal of bacteria and fungi from the denture surface can lead to further biofilm development and prolong the inflammation that may already be present in DS patients. Few *in vitro* models have been developed for studying the disinfection of multispecies denture biofilms, with the majority of these basing their work around the main causative agent *C. albicans* only (Li et al., 2010; Mendonca E Bertolini et al., 2014). Therefore, such models do not represent the complex environment observed clinically in the oral cavity and as such do not investigate the true activity of various denture-cleansing regimens. We have shown the development of a method that not only enables use to rapidly assess the composition of biofilms following antimicrobial challenge, but also to evaluate the viability within these.

Using the model described in this study, we have shown that combinational therapy was the most effective treatment against denture biofilms when compared to monotherapy, in agreement with previous studies (Pellizzaro et al., 2012; Duyck et al., 2016). Of particular interest, the methods used in this study to determine the efficacy of the treatments had strikingly large discrepancies. The measurement of biofilm viability by CFU indicated that denture-cleansing alone was as equally active as combinational treatment against the biofilms, if not even better than DC + B with no growth being detected. However, when this was explored at a more sensitive molecular level using PCR, there appeared to be residual organisms remaining on the PMMA following all denture hygiene regimens, which has significant clinical implications.

PCR is routinely used for identifying and quantifying oral microorganisms (Suzuki et al., 2004a; Park et al., 2011; Millhouse et al., 2014) due to its fast turnaround time as well as its high sensitivity and specificity. However, until recently the technology was not available to distinguish between viable and dead cells, as DNA can persist for an extended period of time following cell death. With the development of a live-dead PCR technique (Nocker and Camper, 2009; Loozen et al., 2011), viable cells can now be distinguished at a more sensitive molecular level, allowing for its use in antimicrobial testing (Sanchez et al., 2013; Sanchez D. A. et al., 2014). This technique proved to be essential in this study when it came to reporting the activity of the denture hygiene regimens, as an overestimation of killing would have been reported based upon culture methodologies alone.

When considering the composition of the biofilms following mechanical and chemical treatment, it is apparent that some organisms within the biofilm are more susceptible to treatment than others. A recent study investigated the prevalence of common periodontal pathogens in elderly patients wearing complete dentures and found that most bacteria including *P. gingivalis* and *P. intermedia* increased over the 6-month observation period, despite satisfactory oral hygiene methods being employed (Andjelkovic et al., 2015). However, in our study, we have shown a significant reduction in both these species using the combination of B + DC, highlighting a more appropriate denture hygiene regimen for this patient group.

What is clear from this study is that *A. naeslundii* and streptococci are the most abundant organisms remaining in the biofilm following denture treatment. These microbes are classed as early colonizers of the oral cavity (Kolenbrander, 2011), with *Streptococcus* species accounting for greater than 60% of the total bacteria colonizing teeth within the first 4 h post-cleaning (Nyvad and Kilian, 1987). Therefore, these organisms can be associated with a “healthy” oral environment and as such it is not of great concern that these are the more predominant species following combinational denture cleaning.

Surprizing from our compositional analysis it is shown that *C. albicans* accounts for only a small proportion of the untreated denture biofilm in comparison to the bacterial species. Previous work has identified the lack of *Candida* adhesion and hyphal formation when in the presence of specific oral bacteria, including *P. gingivalis, Actinomyces* and *Streptococcus* species (Nair and Samaranayake, 1996; Vílchez et al., 2010; Guo et al., 2015), which may explain the reduced number of *C. albicans* cells found in our denture biofilms.

We next aimed to look at the impact of daily combinational treatment and compared this to intermittent cleansing. Based on CFU methodology, daily combinational treatment was superior with complete inhibition of total microbes. This is in contrast to intermittent cleansing whereby re-growth was observed at days 2–4 when no DC was used, concurring with a study carried out by our group previously (Ramage et al., 2012). However, when examined at a molecular level, few differences exist between both hygiene regimens with regards to biofilm viability, with a large number of organisms still persisting post-treatment. This concurs with a recent study where investigators found *C. albicans* can persist despite daily denture cleansing treatment and can allow for proliferation of the residual biofilm (Freitas-Fernandes et al., 2014). Here the authors concluded that daily denture cleansing used in combination with mechanical disruption might improve biofilm disruption.

Although a large number of organisms remained following combinational treatment in our study, specific species were not quantified during the sequential testing and therefore we are unable to determine whether those organisms that remain in the biofilm are non-pathogenic commensals, as identified previously in this study. Moreover, our study highlights the significant discrepancies between culture and molecular methods, emphasizing the importance of employing more than one technique to measure antimicrobial activity.

One explanation to why cells are able to evade therapy is due to the surface roughness of the denture material itself, as this provides an area of pores allowing cells to colonize and escape removal (Li et al., 2010). In our study, we have shown using microscopy techniques, organisms residing in the crevices of the denture material whereby most if not all of which were viable. Although these appear to reduce in number by day 5, they are still not fully eradicated and may be able to proliferate if therapy ceases. This is in agreement with other studies, showing up to 98% reduction of *C. albicans* biofilm viability when treated with other Polident formulations, however, daily usage did not remove residual biomass that remained on the PMMA surfaces (Pellizzaro et al., 2012; Freitas-Fernandes et al., 2014). The 5-day testing period here was a limitation of this study and therefore prolonged treatment times should be considered for full disinfection of the denture biofilm.

Localized inflammation of the oral cavity is not the only concern for effective denture cleansing as there is potential for pathogenic organisms harboring on dentures to disseminate, causing more serious systemic infections in patients (Sumi et al., 2002; Inaba and Amano, 2010; O'donnell et al., 2015b). A recent study by our group identified dentures are a reservoir for respiratory pathogens, concluding these could be the potential source of infection for some cases of aspiration pneumonia (O'donnell et al., 2015b). Furthermore, Sumi et al. found that more than 60% of elderly patients screened had dental plaques colonized with respiratory pathogens (Sumi et al., 2007), another potential reservoir for systemic infections. This concludes that effective cleaning of the oral cavity including good denture hygiene is essential for ensuring localized infections are kept to a minimum.

This study has generated a multispecies denture biofilm model suitable for testing various denture-cleansing regimens. Using this model, it was shown that combinational therapy of brushing and denture cleansing was the most superior oral hygiene regimen for reducing denture biofilm biomass and viability. Furthermore, when treated on a daily basis, the number of viable bacteria and yeast adhered to the PMMA discs was reduced compared to those treated with intermittent cleansing. However, following both treatment regimens, there were still residual organisms found within the crevices of the denture material. While we have not tested an extensive range of cleansers, it is likely that effectiveness of others would also have a similar impact on denture plaque unless their primary mechanism of action was biofilm removal rather than direct antimicrobial activity. Further studies will address the wider impact on denture cleansing regimens. An additional conclusion from this study was that reliance on culture based viability tests is highly inaccurate and more sensitive molecular techniques should be employed for reporting antimicrobial activity in future studies. Moreover, other techniques could be used to supplement this analysis, such as microscopy. This has profound implications for high throughput testing of actives in laboratories not equipped to handle these approaches, and the data generated without this may create an unintentional bias toward the active.

## Author contributions

LS, GL, LO, and EM participated in the study design, carried out the experimental studies, performed statistical analysis and was responsible for the manuscript. CM participated in study design and supervised manuscript writing. OM participated in the study design, undertook the microscopy imaging and analysis, and helped draft the manuscript. CW contributed to study design, data analysis and supervised manuscript writing. DB and AA participated in the study design, analysis of the data and contributed toward the preparation of the manuscript. CN contributed to the study design, data analysis and contributed to the manuscript writing. GR conceived the study, participated in study design and was jointly responsible for writing the final manuscript. All authors read and approved the manuscript.

## Funding

This study was funded by GlaxoSmithKline Oral Health Research & Development (Weybridge, Surrey, UK). LO was funded by the BBSRC CASE studentship (BB/K501013/1). EM was funded through a BBSRC CASE studentship (BB/J500318/1).

## Disclosures

DB and AA are employees of GlaxoSmithKline Consumer Healthcare whose Polident product is evaluated in the testing reported in this study.

### Conflict of interest statement

The authors declare that the research was conducted in the absence of any commercial or financial relationships that could be construed as a potential conflict of interest.
