# Supplementary material for: Viable Compositional Analysis of an Eleven Species Oral Polymicrobial Biofilm
Source: Front Microbiol. 2016 Jun 10;7:912. doi: 10.3389/fmicb.2016.00912 (PMC4902011; doi:10.3389/fmicb.2016.00912)
Supplement: Supplementary file 1 [file Table1.DOCX]

**Supplementary table 1: Percentage of species distribution of treated biofilms**

| **Organism** | **Untreated** | | **DC + B** | | **B + DC** | |
| --- | --- | --- | --- | --- | --- | --- |
|  | **Total (%)** | **Live (%)** | **Total (%)** | **Live (%)** | **Total (%)** | **Live (%)** |
| **AN** | 32.9 | 14.9 | 28.1 | 23.8 | 19.9 | 21.7 |
| **PI** | 3.0 | 6.2 | 0.9 | 1.4 | 0.1 | 0.5 |
| **AA** | 0.2 | 0.7 | 0.3 | 1.2 | 0.1 | 0.9 |
| **VD** | 36.0 | 45.4 | 31.1 | 21.3 | 4.1 | 12.8 |
| **FN** | 14.8 | 12.8 | 23.8 | 13.3 | 20.4 | 27.1 |
| **Strep** | 6.4 | 9.8 | 3.5 | 13.9 | 49.1 | 33.5 |
| **CA** | 0.6 | 0.1 | 4.8 | 0.3 | 5.7 | 0.8 |
| **PG** | 6.0 | 10.1 | 7.5 | 24.6 | 0.6 | 2.7 |
