# Supplementary figures and images for: Viable Compositional Analysis of an Eleven Species Oral Polymicrobial Biofilm
Source: Front Microbiol. 2016 Jun 10;7:912. doi: 10.3389/fmicb.2016.00912 (PMC4902011; doi:10.3389/fmicb.2016.00912)

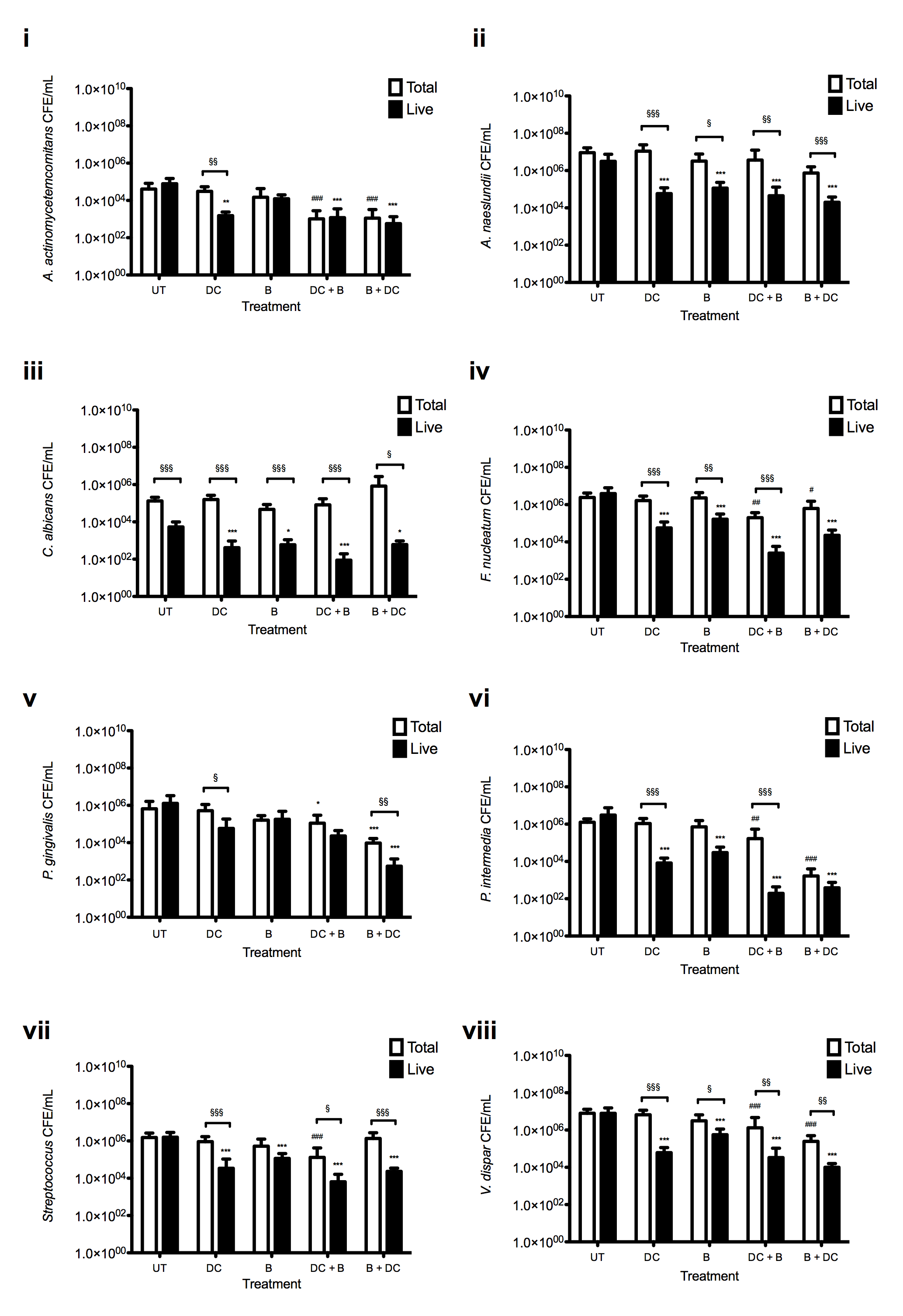

Supplement: Supplementary file 2 [file Image1.TIFF]
