# Supplementary material for: Viable Compositional Analysis of an Eleven Species Oral Polymicrobial Biofilm
Source: Front Microbiol. 2016 Jun 10;7:912. doi: 10.3389/fmicb.2016.00912 (PMC4902011; doi:10.3389/fmicb.2016.00912)
Supplement: Supplementary file 3 [file DataSheet1.DOCX]

**Supplementary figure 1: Impact of denture cleansing therapies on specific bacteria and fungi within denture biofilms.** Complex 11 species biofilms were grown on PMMA for 7 days as described previously, before treated with the four therapies; denture-cleansing (DC), brushing (B), cleansing then brushing (DC + B) and brushing then cleansing (B + DC). Following treatment, each disc was sonicated before 50 μM of PMA was added and exposed to a 650 w halogen light source for 5 min to allow photo activation. Samples containing no PMA were also included to account for total biomass. DNA was extracted from each sample using the Qiagen DNA extraction kit, for quantification of each species using SYBR^®^ GreenER^™^ based qPCR to determine the number of total and live cells remaining following treatment. Total biomass and live cells for A.A (**i**), A.N (**ii**), C.A (**iii**), F.N (**iv**), P.G (**v**), P.I (**vi**), Strep (**vii**) and V.D (**viii**) to show the differences between each species. Untreated and uninoculated controls were also included. All testing was carried out in triplicate and on three independent occasions. Data represents mean ± SD, statistical analysis of treatments was compared to their respective untreated controls, in addition to total versus live for each therapy (*/^#^/^§^p<0.05, **/^##^/^§§^p<0.01, ***/^###^/^§§§^p<0.001).
